# Effect of Prenatal Alcohol Exposure on Childhood Academic Outcomes: Contrasting Maternal and Paternal Associations in the ALSPAC Study

**DOI:** 10.1371/journal.pone.0074844

**Published:** 2013-10-09

**Authors:** Rosa Alati, George Davey Smith, Sarah J. Lewis, Kapil Sayal, Elizabeth S. Draper, Jean Golding, Robert Fraser, Ron Gray

**Affiliations:** 1 School of Population Health, University of Queensland, Brisbane, Queensland, Australia; 2 Centre for Youth Substance Abuse Research, University of Queensland, Brisbane, Queensland, Australia; 3 School of Social and Community Medicine, University of Bristol, Bristol, United Kingdom; 4 MRC Integrative Epidemiology Unit, University of Bristol, Bristol, United Kingdom; 5 Developmental Psychiatry, University of Nottingham, Nottingham, United Kingdom; 6 Department of Health Sciences, University of Leicester, Leicester, United Kingdom; 7 Reproductive and Developmental Medicine, University of Sheffield, Sheffield, United Kingdom; 8 National Perinatal Epidemiology Unit, University of Oxford, Oxford, United Kingdom; The University of Texas M. D. Anderson Cancer Center, United States of America

## Abstract

**Background:**

The impact of low-to-moderate levels of alcohol consumption during pregnancy on child cognitive outcomes has been of recent concern. This study has tested the hypothesis that low-to-moderate maternal alcohol use in pregnancy is associated with lower school test scores at age 11 in the offspring via intrauterine mechanisms.

**Methods:**

We used data from the Avon Longitudinal Study of Parents and Children (ALSPAC), a birth cohort study based in the South West of England. Analyses were conducted on 7062 participants who had complete data on: maternal and paternal patterns of alcohol use in the first trimester and at 18 weeks' gestation, child's academic outcomes measured at age 11, gender, maternal age, parity, marital status, ethnicity, household crowding, home ownership status and parental education. We contrasted the association of mother's alcohol consumption during pregnancy with child's National Curriculum Key Stage 2 (KS2) test scores with the association for father's alcohol consumption (during the time the mother was pregnant) with child's National Curriculum Key Stage 2 (KS2) test scores. We used multivariate linear regression to estimate mean differences and 95% confidence intervals [CI] in KS2 scores across the exposure categories and computed *f* statistics to compare maternal and paternal associations.

**Findings and conclusions:**

Drinking up to 1 unit of alcohol a day during pregnancy was not associated with lower test scores. However, frequent prenatal consumption of 4 units (equivalent to 32 grams of alcohol) on each single drinking occasion was associated with reduced educational attainment [Mean change in offspring KS2 score was −0.68 (−1.03, −0.33) for maternal alcohol categories compared to 0.27 (0.07, 0.46) for paternal alcohol categories]. Frequent consumption of 4 units of alcohol during pregnancy may adversely affect childhood academic outcomes via intrauterine mechanisms.

## Introduction

Excessive alcohol use in pregnancy can lead to a range of physical, behavioural and cognitive sequelae in the child, generally known as Fetal Alcohol Spectrum Disorder (FASD) [1], [2], [3]. Many such children display a wide variety of neurological impairments confirmed by both neuroimaging and neuropsychological studies [4], [5]. Some researchers also claim that even low-to-moderate alcohol use in pregnancy can cause neuropsychological impairments without any evidence of physical abnormality [6], [7], [8]. Findings from the Seattle Prospective Longitudinal Study have repeatedly demonstrated associations between all levels of alcohol consumed in pregnancy and attention problems, lower IQ and problems with spatial-visual memory [8]. In the Pittsburgh study of prenatal substance use, moderate prenatal alcohol exposure was also found to predict decreased cognitive function at age 10 among African American offspring [9]. In an African American cohort of children born in Detroit, those exposed to low levels of alcohol in pregnancy displayed neuropsychological deficits, particularly in attention, learning and cognitive flexibility, when compared with those with no prenatal exposure to alcohol [6], [7].

In contrast, others have argued that the existing evidence linking neuro-developmental disabilities with low-to-moderate alcohol exposure in pregnancy is not conclusive [10], [11], [12], [13], [14]. Findings from large population based birth cohort studies suggest no increased risk of adverse neuro-developmental outcomes in children of mothers who consumed low-to moderate levels of alcohol in pregnancy [10], [15], [16], [17], [18] Three studies using data from the Millennium Cohort Study suggest that offspring of light drinkers may be at a lower risk of neuro-behavioural problems compared with offspring of abstainers [15], [16], [17]. These studies suggest that offspring born of mothers who drank up to 2 drinks a week, or per occasion, had similar if not higher cognitive test scores compared with offspring born to abstainers [15], [16], [17] Though it has been noted that the poorer socio-educational background of those in the abstaining group may explain the association with the offspring's cognitive outcomes, these studies have led to heated debate [19], [20] as public health concern regarding alcohol use in pregnancy grows.

In this analysis we aim to evaluate the effects of confounding by contrasting the effects of mother's alcohol use during pregnancy on child's academic scores with the effects of father's alcohol use (also measured during the time of the pregnancy) [21], [22], [23] with child's academic scores. If the biological effects of maternal alcohol consumption in pregnancy directly impact on child's academic outcomes, we would expect maternal effects on child's academic outcomes through an intrauterine mechanism to be much stronger than, or discordant from, paternal effects. If, on the contrary, there are similar parental associations of alcohol use with child's academic outcomes, it is likely that observed maternal effects are due to background factors not adequately identifiable through traditional statistical adjustment [24]. In this study we used a comparison of the effect of maternal and paternal exposure to alcohol use measured during the time period of the pregnancy, to test associations of in-utero exposure to alcohol use with offspring's academic outcomes, measured by National Curriculum Key Stage 2 (KS2) scores at child age 11.

## Methods

We used data from the Avon Longitudinal Study of Parents and Children (ALSPAC). ALSPAC is a birth cohort study of children born to women who were resident in the former Avon region in the South West of England while pregnant. The overarching aim of the ALSPAC study was to investigate modifiable influences on child health and development [25] All pregnant women residing in the area, who were due to give birth between April 1991 and December 1992 (n = 14,541). Of these 13,922 (95%) had a singleton, live born child were enrolled in the study. ALSPAC recruitment and data collection strategies are fully described elsewhere (http://www.alspac.bris.ac.uk/welcome/index.shtml) [25].

### Ethical statement

Participants were told that taking part in the study was voluntary, that their questionnaire information would be linked to other information and that if they did not want this to happen they could opt out at any time. This procedure met the ethical standards at the time mothers were first interviewed, and ethics approval for the study was obtained from the ALSPAC Law and Ethics Committee and the Local Research Ethics Committees. Further discussion on the ethical aspects of the ALSPAC study can be found elsewhere [26]. The sample for the present study includes all singleton babies still alive at 1 year of age, for whom relevant data were available in pregnancy, including at 18–23 weeks gestation, birth, 8 weeks after the birth and from the National Curriculum Key Stage 2 tests performed at age 11.

### Measurements

#### Maternal and paternal alcohol use

Self reports of alcohol use from mothers and their partners were used to contrast maternal and paternal associations with offspring cognitive abilities at age 11. Two measures of alcohol use were available at 18 weeks of gestation. Mothers and their partners were first asked how often they had consumed alcoholic drinks during the first 3 months of pregnancy (Never, Less than 1 glass a week, At least 1 glass a week, 1 or 2 glasses every day, At least 3–9 glasses every day, and At least 10 glasses every day). The following footnote was included ‘by glass we mean a pub measure of spirits, half a pint of lager or cider, a wine glass of wine’. Examples explained that one drink was equivalent to one UK unit of alcohol, corresponding to 8 grams of alcohol. The variable was re-coded into: Never, <1 glass a week, 1–6 a week, 1+ a day. Mothers and their partners were also asked how many days in the previous month they had consumed the equivalent of 2 pints of beer, 4 glasses of wine or 4 pub measures of spirit or more. The response was coded as never, 1–2 days, 3–4 days, 5–10 days, 10+ days, everyday, which was re-coded to never, 1–4 days, 5–10 days, 10+ days. This question aimed to identify consumption of larger volumes of alcohol on each single drinking occasion. Though this drinking pattern described here does not necessarily involve levels of use that meet definitions for binging or binge drinking (there is international variation in definitions) [27], [28], [29], we refer to it as ‘binge’ patterns of drinking to distinguish it from the regular but lighter daily consumption not exceeding one drink a day. We also used maternal reports at 8 months after the birth to compare maternal prenatal and postnatal alcohol consumption.

#### Academic outcomes

In the United Kingdom pupils aged between 7 and 11 follow Key Stage 2 of the National Curriculum from Years 3 through to Year 6. At the end of this stage in year 6, pupils aged 11 are tested as part of the program of National Curriculum Tests, which cover English, Mathematics and Science. Key Stage 2 (KS2) scores provide records of attainment in these subjects and are considered to be a ‘real world’ measure of academic performance. The tests are externally marked, with results for each school being published in performance tables. Further details are available at http://curriculum.qcda.gov.uk/key-stages-1-and-2/index.aspx. A data linkage conducted with the Department for Children, Schools and Families provided KS2 scores data for 11,974 ALSPAC children attending publically funded schools in England. KS2 scores were available as a standardised, age-adjusted total score for English, Maths and Science.

#### Potential confounders

Indicators of socio-economic position were obtained by maternal self-reports in pregnancy. They included maternal marital status (never married, widowed/divorced/separated, married/cohabiting), home ownership (mortgaged/owned/rented), an index indicating the crowding condition of the household (calculated by dividing the number of people in the household by the number of rooms, < = 0.5, 0.5–0.75, 0.75−>1.0), and ethnicity (white, not-white). Gender of the child was extracted from the official birth notifications. Maternal parity (no. of previous pregnancies resulting in a live or still-birth: 0, 1–2, 3+) was obtained at 18 weeks' gestation, highest maternal and paternal educational qualifications obtained at 32 weeks gestation (Degree, A levels, O levels, Vocational/CSE). Smoking status was assessed by asking mothers and their partners how often they smoked in the first 3 months of pregnancy and dichotomised (‘non smoker’ and ‘smoker’). We used maternal reports of their partners' tobacco use and education, as they showed high agreement with partners' own self-reports available on a subsample of the cohort (Kendall's τ_b_ were 0.91 and 0.82 for tobacco use and education respectively).

### Statistical analyses

We presented the means and standard deviations of child's KS2 scores by categories of maternal and paternal alcohol consumption for comparison. We used linear regression models to estimate mean differences and 95% confidence intervals [CI] in KS2 scores across the exposure categories. Then we fitted multivariate linear regression models to assess the independent effect per category of alcohol on academic abilities. We adjusted for paternal effects in the maternal model and maternal effects in the paternal model, sex (Model 1), in addition, parity, ethnicity, home ownership and crowding (Model 2), plus maternal and paternal education (Model 3) and finally all the above plus maternal and paternal smoking (Model 4). We computed an *f* statistic to formally compare the coefficients of maternal and paternal associations for each model. Then, we compared maternal prenatal and postnatal alcohol consumption on child's KS2 scores. Finally we conducted two additional analyses. In one analysis, we explored whether maternal depression in pregnancy attenuated associations between maternal and paternal alcohol consumption and KS2 scores. In another analysis we investigated biological paternity by excluding 79 fathers for whom biological paternity was uncertain.

Complete case analyses presented in this paper were conducted on 7062 study participants (about 54% of the eligible live born singleton children who were still alive at 1 year of age) with complete data on maternal and paternal alcohol consumption at baseline, offspring KS2 scores at age 11 and all confounding variables. KS2 data available on 11974 participants (90% of the original cohort sample) allowed us to examine associations between our main exposures and confounders and academic outcomes in those who would be otherwise classed as lost to follow-up. A standard comparison between participants still in the study and those lost to follow-up was also conducted. All analyses were conducted using STATA 12 [30].

## Results

Table 1 compares participants with complete data on all variables (complete dataset) with participants for whom KS2 scores were available from the linked data (incomplete dataset with KS2 data). Attrition was differential for the majority of the predictors and confounders of interest. Children with lower KS2 scores were under-represented in the ‘complete dataset’ group as were those born of mothers who reported drinking alcohol daily and having several alcoholic drinks a day. Offspring of mothers who were unmarried, had more children, lower levels of education and lived in rented or crowded accommodation were also under-represented in the ‘complete dataset’ group. The only deviation from this pattern of missingness was paternal daily and ‘binge’ patterns of alcohol consumption. These patterns of alcohol consumption were very similar in the complete case group and those lost to the study (data not shown).

**Table 1:** Comparisons between those with complete data and those with KS2 data but incomplete data on demographic and other variables.

		Complete dataset	Incomplete dataset (KS2 data only)
	N	Mean [SD]	Mean [SD]
**KS2 score**	11,974	7,062	4,912
		102.4 [8.9]	98.4 [10.4]
	**N**	**%**	**%**
**sex**	11,974	7,062	4,912
male	6097	50.6	51.4
female	5877	49.4	48.6
*χ ^2^ = 0.66 p = 0.42*			
**Marital Status**	11,273	7,062	4,912
married	8521	81.9	65.1
never married	2111	13.6	27.3
wid/separated	641	4.5	7.6
*χ ^2^ = 407.21 p = 0.0001*			
**Ethnicity**	10,663	7,062	3,601
white	10419	98.5	96.2
other	244	1.5	3.8
*χ ^2^ = 55.90 p = 0.0001*			
**Parity**	11,141	7062	4,079
none	4896	46.2	40.0
1–2	5566	49.0	51.7
3+	679	4.8	8.3
*χ ^2^ = 75.91 p = 0.0001*			
**Home mortgage/ownership**	11,214	7062	4,152
Yes	8392	80.8	64.7
rented	2822	19.2	35.3
*χ ^2^ = 390.11 p = 0.0001*			
**House crowding**	11,053	7062	3,991
< = 0.5	4555	45.6	33.5
>0.5–0.75	5783	49.6	57.1
0.75->1.0	715	4.8	9.4
*χ ^2^ = 211.28 p = 0.0001*			
**Parental education**	10,721	7062	3,659
Degree	1723	19.3	9.9
A levels	2808	27.9	22.8
O levels	2564	24.7	22.5
Vocational/CSE	3626	28.2	44.7
*χ ^2^ = 354.93 p = 0.0001*			
**Maternal Age**	11,974	7,062	4,912
15–19	535	2.8	6.9
20–29	7928	65.0	68.0
30–39	3371	31.2	23.8
40+	140	1.1	1.3
*χ ^2^ = 169.38 p = 0.0001*			
**Maternal smoking**	11279	7062	4217
no smoker	8545	80.0	68.6
smoker	2734	20.0	31.4
*χ ^2^ = 187.86 p<0.001*			
**Paternal smoking**	*10542*	*7062*	4217
no smoker	6798	68.1	57.2
smoker	3744	31.9	42.8
*χ ^2^ = 187.86 p<0.001*			
**Maternal regular drinking**	11,204	7,062	4142
never	5105	45.7	45.4
<1 glass a week	4370	40.1	37.2
1–6 a week	1524	12.8	15.0
1+ pday	205	1.5	2.5
*χ ^2^ = 29.01 p<0.001*			
**Maternal ‘binge’ patterns of drinking**	11,158	7062	4,096
none	9262	84.6	80.3
1–4 days	1433	11.9	14.6
5–10 days	223	1.8	2.4
10+ days	240	1.8	2.7
*χ ^2^ = 38.35 p<0.001*			
**Paternal regular drinking**	8,379	7,062	1,317
never	374	3.9	7.4
<1 glass a week	2098	24.6	27.3
1–6 a week	4268	51.8	46.4
1+ pday	1639	19.7	18.9
*χ ^2^ = 39.44 p<0.001*			
**Paternal ‘binge’ patterns of drinking**	8,454	7,062	1,392
none	1496	17.5	18.5
1–4 days	3189	37.7	38.1
5–10 days	2153	25.7	24.4
10+ days	1616	19.1	19.0
*χ ^2^ = 1.45 p = 0.694*			

Tables 2 and 3 show univariable associations (means and standard deviation) between maternal and paternal patterns of alcohol use during pregnancy (table 2), potential confounders (table 3) and child's academic outcomes measured by KS2 scores at age 11. There were positive associations between regular alcohol consumption and child's KS2 scores and inverse associations between frequency of maternal alcohol consumption of 4 or more units and child's KS2 scores. By comparison, there was a positive association between paternal regular alcohol consumption and child's KS2 scores, and no associations between paternal ‘binge’ patterns of alcohol consumption and child's scores. All indicators of parental socio-economic disadvantage were associated with lower KS2 scores in the offspring (Table 3). A summary of potential confounders in relation to maternal and paternal alcohol consumption is presented in Table S1 and Table S2.

**Table 2:** Univariable associations between maternal and paternal patterns of alcohol consumption during pregnancy and child's KS2 scores [means (SD)] at age 11.

Maternal regular alcohol consumption					
		N	Means	[SD]	p*
**KS2 score**					
	never	3332	102.0	[9.1]	<0.001
	<1 glass a week	2928	102.8	[8.7]	
	1–6 a week	947	102.7	[8.7]	
	1+ a day	106	100.3	[10.7]	
**Paternal regular alcohol consumption**					
**KS2 score**	never	285	98.9	[11.0]	<0.001
	<1 glass a week	1791	101.1	[9.1]	
	1–6 a week	3786	102.8	[8.7]	
	1+ a day	1451	103.5	[8.5]	
**Maternal ‘binge’ patterns of drinking (more than 4 units)**					
**KS2 score**	None	6181	102.7	[8.9]	<0.001
	1–4 days	873	100.8	[9.1]	
	5–10 days	126	100.9	[8.2]	
	10+ days	133	99.4	[9.3]	
**Paternal ‘binge’ patterns of drinking (more than 4 units)**					
**KS2 score**					
	None	1264	101.8	[9.4]	= 0.071
	1–4 days	2754	102.2	[8.7]	
	5–10 days	1890	102.8	[8.5]	
	10+ days	1405	102.6	[8.5]	

* p for linear association.

**Table 3:** Univariable associations between KS2 scores and potential confounding factors [means (SD)] at age 11 (complete case analysis, n = 7062)].

Gender	KS2 score				
	N	Means	[SD]	p*	
Male	3574	102.0	[9.1]	<0.001	
Female	3488	102.7	[8.6]		
**Marital status**					
Married	5781	102.9	[8.5]	<0.001	
Never Married	961	100.0	[10.3]		
Sep/divorced	320	100.8	[9.4]		
**Ethnicity**					
Caucasian	6955	102.4	[9.0]		0.087
other	107	103.9	[9.8]		
**Parity**					
0	3263	103.4	[8.5]		<0.001
1–2	3457	101.8	[9.0]		
3+	342	99.2	[10.3]		
**Home mortgage/ownership**					
mortgaged/owned	5705	103.3	[8.3]		<0.001
rented	1357	98.5	[10.2]		
**House crowding**					
< = 0.5	3220	104.3	[8.0]		<0.001
>0.5–0.75	3504	101.2	[9.2]		
0.75−>1.0	338	97.4	[10.0]		
**Maternal and paternal education**					
Degree	1360	107.8	[6.5]		<0.001
A levels	1972	103.4	[7.7]		
O levels	1741	101.9	[8.6]		
Vocational/CSE	1989	98.2	[9.5]		
**Maternal age**					
31–44 yrs	2277	104.2	[8.4]		<0.001
20–30 yrs	4464	101.9	[8.8]		
20 or less	321	97.1	[10.2]		
**Maternal smoking**					
non smoker	5652	103.0	[8.6]		<0.001
smoker	1410	100.0	[9.6]		
**Paternal smoking**					
non smoker	4808	103.4	[8.6]		<0.001
smoker	2254	100.4	[9.2]		

* p for linear association.

**Figure 1:**
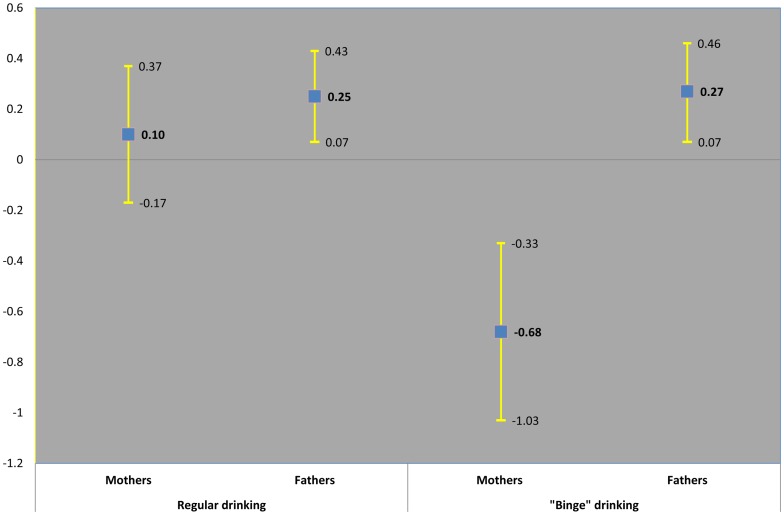
Mean change in offspring KS2 score per increase in maternal and paternal alcohol categories. Adjusted for sex, maternal age, parity, socio-economic position, ethnicity, maternal and paternal education and smoking and other parent's alcohol consumption.

**Figure 2:**
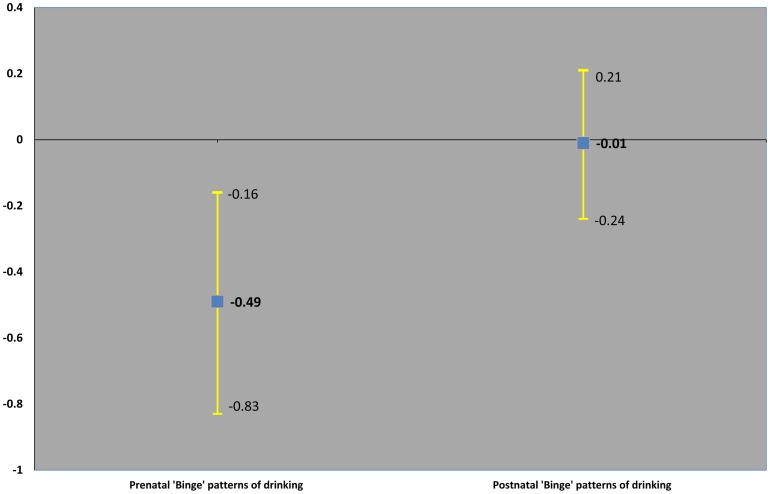
Mean change in offspring KS2 score per increase in maternal alcohol categories during and after pregnancy. Adjusted for sex, maternal age, parity, socio-economic position, ethnicity, maternal and paternal education and smoking and other parent's alcohol consumption.

Table 4 and Figure 1 show complete case analysis for the mean change in offspring KS2 scores per increase in maternal and paternal categories of alcohol use, and the p-value for the difference between maternal and paternal associations. The positive association of paternal regular consumption and offspring KS2 scores attenuated towards the null with adjustment for parental education. The association of more frequent maternal alcohol consumption of 4 or more units with lower KS2 scores remained after progressive adjustment for socio-economic position and parental education (Figure 1). In the fully adjusted models there was statistical evidence of a difference in the association between maternal ‘binge’ drinking-offspring KS2 scores compared with paternal ‘binge’ drinking-offspring KS2 scores [Mean change in offspring K2 score was −0.68 (−1.03, −0.33) for maternal alcohol categories compared to 0.27 (0.07, 0.46) for paternal alcohol categories]. We found no evidence of non linearity in maternal or paternal models (p-value for likelihood ratio test were 0.6091 and 0.6911 respectively for maternal and paternal models). In a sensitivity analysis testing the effect of maternal prenatal depression on a reduced sample of 6593 participants (data not shown), the mean change in offspring KS2 score was slightly attenuated for maternal ‘binge’ drinking categories (– 0.56 (95% CI −0.92, −0.19), but the same for paternal ‘binge’ drinking categories (0.26 (95% CI 0.06, 0.47). The evidence of a difference in the maternal – paternal comparisons remained (p-value for the *f* statistic  = 0.0003]. Finally, table 5 and Figure 2 show comparisons of maternal prenatal and postnatal ‘binge’ drinking with KS2 scores. The negative association of maternal ‘binge’ drinking with KS2 scores was stronger for prenatal than postnatal ‘binge’ patterns of alcohol use measured 8 months after the birth period, with statistical evidence of a difference (Figure 2). Additional analyses excluding 79 fathers for whom biological paternity was uncertain yielded the same findings reported here (data not shown).

**Table 4:** Mean change in offspring KS2 score per increase in maternal and paternal alcohol categories.

	Mean change in offspring KS2 score per increase in maternal alcohol categories	Mean change in offspring KS2 score per increase in paternal alcohol categories	p-value for the difference between maternal and paternal associations	
Adjustments	Complete case analyses (n = 7062)			
	**Regular alcohol consumption in first 3 months of pregnancy**			
Adjusted for sex and other parent's alcohol consumption	0.06 (−0.21, 0.34)	0.84 (0.65, 1.03)	<0.001	
Plus maternal age, parity, socio-economic position and ethnicity	0.14 (0.32, 0.00)	0.44 (0.26, 0.62)	= 0.030	
Plus maternal and paternal education	0.08 (−0.18, 0.34)	0.25 (0.07, 0.42)	= 0.334	
Plus maternal and paternal smoking	0.10 (−0.17, 0.37)	0.25 (0.07, 0.43)	= 0.406	
	**‘Binge’ patterns of drinking during pregnancy**			
Adjusted for sex and other parent's alcohol consumption	−1.43 (−1.80, −1.06)	0.44 (0.23, 0.65)		<0.0001
Plus maternal age, parity, socio-economic position and ethnicity	−0.98 (−1.33, −0.62)	0.24 (0.04, 0.44)		<0.0001
Plus maternal and paternal education	−0.72 (−1.06, −0.38)	0.24 (0.05, 0.43)		<0.0001
Plus maternal and paternal smoking	−0.68 (−1.03, −0.33)	0.27 (0.07, 0.46)		<0.0001

**Table 5:** Mean change in offspring KS2 score per increase in maternal alcohol categories during and after pregnancy.

	Mean change in offspring KS2 score per increase in maternal prenatal alcohol categories	Mean change in offspring KS2 score per increase in maternal postnatal alcohol categories	p-value for the difference between prenatal and postnatal associations
Adjustments	Prenatal ‘Binge’ patterns of drinking	Postnatal ‘Binge’ patterns of drinking (8 months after the birth)	*P*
Adjusted for sex and ‘binge’ drinking at the other time point	−1.27 (−1.62, −0.91)	0.20 (−0.04, 0.44)	<0.0001
Plus maternal age, parity, socio-economic position and ethnicity	−0.79 (−1.13, −0.45)	0.00 (−0.23, 0.24)	<.0.001
Plus maternal and paternal education	−0.52 (−0.85, −0.20)	−0.04 (−0.26, 0.18)	= 0.033
Plus maternal and paternal smoking	−0.49 (−0.83, −0.16)	−0.01 (−0.24, 0.21)	= 0.038

## Discussion

Studies reporting the effects of prenatal alcohol exposure have yielded conflicting results. A major challenge to advancing research in this area has been accounting for confounding factors which could explain observed links between maternal alcohol use in pregnancy and child offspring outcomes [31], [32]. In this paper, we used a parental-offspring comparison analysis to address this issue and test whether associations between maternal alcohol use during pregnancy and offspring's academic abilities could be better explained by likely biological mechanisms than by effects of residual confounding from social and lifestyle factors. We hypothesised that contrasting associations of maternal and paternal alcohol consumption with child's academic abilities would point to an intra-uterine influence of maternal alcohol use during pregnancy, as there is no strong evidence of intra-uterine mechanisms linking paternal alcohol use with offspring cognitive development.

More frequent consumption by the mother of 4 or more units of alcohol (an equivalent of 32 grams of alcohol) per single drinking occasion during pregnancy was associated with lower academic abilities in offspring in this study. Associations between the effect of 6 or more drinks per occasion and lower cognitive function have been previously reported [7], [33] but here we have demonstrated that effects can be seen at lower levels of consumption. Four units equate to around 32 grams of alcohol equivalent to about 2 pints of beer or 2 glasses of wine served at bars or restaurants in the UK. This amount would not necessarily be considered as ‘binging’ but more likely as moderate drinking during a single drinking occasion. Therefore it could be argued that our findings provide support for the claim that moderate drinking during pregnancy is in fact detrimental to child academic outcomes, since greater frequency of this pattern of alcohol consumption by mothers was associated with lower KS2 scores in children at age 11, in contrast with the same levels of alcohol consumption by their partners, which was associated with higher KS2 scores. Additional analyses comparing this pattern of maternal alcohol use before and after the pregnancy on child's KS2 scores found stronger effects during the prenatal period, which is consistent with our main analysis. Future investigations using more sensitive information on volumes of alcohol consumed on each drinking occasion are needed to replicate our findings and further our understanding of the level of risk associated with this pattern of alcohol use.

Maternal consumption of up to one unit per day was not associated with lower academic abilities in this analysis. In a recent study that used genetic variation in alcohol metabolising genes to address confounding, this team has found evidence that four genetic variants associated with alcohol metabolism were related to differences in child's IQ at age 8 in a population of women who drank moderately [34]. This technique of using genotype to obtain unconfounded estimates is known as Mendelian Randomization [24], [35], [36]. The lack of evidence of an effect of regular levels of alcohol exposure in our study may be due to the different outcomes investigated (IQ in the Lewis et al study versus a measure of academic achievement in this study) or the use of a potentially more sensitive technique in the Lewis et al study. Further studies are necessary to investigate the effects of consuming up to one unit daily on other neuropsychological outcomes at different ages.

Few studies have adopted similar robust designs to investigate the effects of in-utero alcohol exposure on developmental outcomes [10], [37]. Our comparison analysis suggests that at least for the patterns of alcohol use described here (more frequent consumption by the mother of 4 or more units), the teratogenic action of ethanol on the developing brain may lead to adverse cognitive and developmental outcomes in children via a intra-uterine mechanism [38]. Using sibling-pair analysis, D'Onofrio and colleagues suggest causal inference between greater prenatal alcohol exposure and conduct problems at ages 4 to 11 [37]. Our study is in line with these findings as it suggests a direct link between the patterns of alcohol consumption in pregnancy described here and offspring academic abilities. In a previous study where we compared maternal versus paternal consumption of alcohol, we did not find strong evidence of any patterns of prenatal alcohol use on child IQ [10]. However also in that study there was a suggestion of negative estimates for mothers' alcohol use compared with positive estimates for father's alcohol use [10]. Therefore, it is possible that our different conclusions are due to our early study being based on a restricted sample of children and having limited power to detect a difference, rather than being due to true dissonance in the findings.

Our findings should be viewed in the context of some limitations. Most analyses presented here have been conducted on 54% of the original cohort sample. Such levels of attrition may introduce bias in our results. We were able to compare those with complete data on all variables of interest to those with complete KS2 scores at age 11 but incomplete data on other covariates. With most studies, this is not possible since both predictor and outcome data are missing, but in this case, availability of linked, complete data on KS2 scores allowed us to observe differences in KS2 scores between those with complete data and those with KS2 only data as well as associations between exposure to alcohol use and KS2 scores in those for whom exposure and other covariate data was missing. Comparisons between those with complete data and those with KS2 data, but incomplete data on other variables, showed that children with lower KS2 scores and children of mothers who drank more often were more likely to be in the ‘incomplete data’ group, compared to those who did not. These overall trends suggest that any attrition in the study may have underestimated the association we found between maternal alcohol consumption and KS2 scores.

This study also has considerable strengths, the most notable of which are the large sample size on which the analysis was conducted and the availability of paternal data on alcohol use during pregnancy. This has provided us with a unique opportunity to conduct parent-child association analyses using one of the best established longitudinal resources in the world, the ALSPAC study, involving a large cohort of children, followed up from pregnancy to age 11 with comprehensive data collected at several time periods.

A number of countries have proposed advice or guidelines for pregnant women on alcohol consumption during pregnancy. Guidelines for Australia and other English-speaking countries are reviewed by O'Leary et al [39]. In the United Kingdom the guidelines were changed in 2009 to advise pregnant women to avoid drinking alcohol. If they did choose to drink to consume no more than 1 to 2 units of alcohol (8–16grams) once or twice a week. Taking our findings in this study together with other studies from the ALSPAC cohort on prenatal alcohol exposure we would advise a precautionary approach until more is known [40]. Even if it becomes possible to find a threshold for harm at average levels of consumption, it is clear that individual susceptibility varies in association with both genetic and environmental factors [34]. Hence the need for cautious public health messages in this area.

In conclusion, this study presents some of the most compelling evidence to date that a pattern of 4 or more units of alcohol consumed on each single drinking occasion by mothers in early pregnancy may influence academic abilities in their offspring at age 11 via intra-uterine effects.

## Supporting Information

Table S1 **Associations between maternal and paternal alcohol consumption in the 1st 3 months of pregnancy and potential confounding factors (complete case analysis (n = 7062).**
(DOCX)

Table S2 **Associations between maternal and paternal ‘binge’ patterns of drinking in the 1st 3 months of pregnancy and potential confounding factors (complete case analysis (n = 7062).**
(DOCX)
