# Supplementary material for: Effect of Prenatal Alcohol Exposure on Childhood Academic Outcomes: Contrasting Maternal and Paternal Associations in the ALSPAC Study
Source: PLoS One. 2013 Oct 9;8(10):e74844. doi: 10.1371/journal.pone.0074844 (PMC3794033; doi:10.1371/journal.pone.0074844)
Supplement: Table S1 — Associations between maternal and paternal alcohol consumption in the 1st 3 months of pregnancy and potential confounding factors (complete case analysis (n = 7062). (DOCX) [file pone.0074844.s001.docx]

**Table S1: Associations between maternal and paternal alcohol consumption in the 1st 3 months of pregnancy and potential confounding factors (complete case analysis (n=7062)**

|  | **Maternal regular alcohol consumption** | | | | | |  | **Paternal regular alcohol consumption** | | | | |  |
| --- | --- | --- | --- | --- | --- | --- | --- | --- | --- | --- | --- | --- | --- |
|  |  | **never** | **< 1 glass a week** | **1-6 a week** | **1 + pday** | **Total** |  | **never** | **<1 glass a week** | **1-6 a week** | **1 + pday** | **Total** |  |
|  | N= | (3224) | (2831) | (904) | (103) | (7062) |  | (277) | (1738) | (3657) | (1390) | (7062) |  |
|  | **sex** | % | % | % | % | N |  | % | % | % | % | N |  |
|  | male | 45.5 | 39.7 | 13.3 | 1.6 | 3574 |  | 4.3 | 24.5 | 51.2 | 20.0 | 3574 |  |
|  | female | 45.8 | 40.5 | 12.3 | 1.3 | 3488 |  | 3.6 | 24.8 | 52.4 | 19.3 | 3488 |  |
|  |  | *χ ² = 1.74, p = 0.55* | | |  |  |  | *χ ² = 3.32, p = 0.34* | | |  |  |  |
|  | **Marital Status** | |  |  |  |  |  |  |  |  |  |  |  |
|  | married | 46.1 | 41 | 11.8 | 1.1 | 5781 |  | 3.5 | 24.2 | 52.8 | 19.6 | 5980 |  |
|  | never married | 46.9 | 34.5 | 16.1 | 2.4 | 961 |  | 6.2 | 26.7 | 47.3 | 19.9 | 1001 |  |
|  | wid/separated | 33.1 | 40.3 | 21.3 | 5.3 | 320 |  | 4.5 | 23.8 | 47 | 24.7 | 332 |  |
|  |  | *χ ² = 94.79 p <0.001* | | |  |  |  | *χ ² = 29.64 p < 0.001* | | |  |  |  |
|  | **Ethnicity** |  |  |  |  |  |  |  |  |  |  |  |  |
|  | white | 45.5 | 40.1 | 12.9 | 1.5 | 6955 |  | 3.7 | 24.5 | 52.1 | 19.6 | 6955 |  |
|  | other | 54.2 | 38.3 | 6.5 | 0.9 | 107 |  | 16.8 | 31.8 | 29 | 22.4 | 107 |  |
|  |  | *χ ² = 5.36 p = 0.15* | | |  |  |  | *χ ² = 59.70 p < 0.001* | | |  |  |  |
|  | **Parity** |  |  |  |  |  |  |  |  |  |  |  |  |
|  | none | 49.4 | 36.9 | 12 | 1.7 | 3263 |  | 3.7 | 21.9 | 53.8 | 20.5 | 3263 |  |
|  | 1-2 children | 42.6 | 43.3 | 13 | 1.1 | 3457 |  | 3.6 | 26.4 | 50.9 | 19.1 | 3457 |  |
|  | 3+ children | 41.5 | 38 | 18.1 | 2.3 | 342 |  | 8.5 | 32.5 | 41.2 | 17.8 | 342 |  |
|  |  | *χ ² = 51.19 p <0.001* | | |  |  |  | *χ ² = 54.07 p < 0.001* | | |  |  |  |
|  | **Home mortgage/ownership** | | |  |  |  |  |  |  |  |  |  |  |
|  | Yes | 45.3 | 40.9 | 12.6 | 1.3 | 5705 |  | 3.2 | 23.1 | 53.4 | 20.3 | 5705 |  |
|  | rented | 47.2 | 36.8 | 13.9 | 2.1 | 1357 |  | 7 | 31 | 45 | 17.1 | 1357 |  |
|  |  | *χ ² = 12.00 p = 0.007* | | |  |  |  | *χ ² = 88.83 p <0.001* | | |  |  |  |
|  | **House crowding** | |  |  |  |  |  |  |  |  |  |  |  |
|  | <=0.5 | 46.9 | 38.7 | 13 | 1.4 | 3220 |  | 2.4 | 19.7 | 56.1 | 21.8 | 3220 |  |
|  | >0.5-0.75 | 44.6 | 41.3 | 12.8 | 1.3 | 3504 |  | 4.7 | 28.5 | 48.9 | 18 | 3504 |  |
|  | 0.75->1.0 | 45.3 | 40.5 | 10.7 | 3.6 | 338 |  | 10.4 | 31.7 | 41.1 | 16.9 | 338 |  |
|  |  | *χ ² = 17.07 p = 0.009* | | |  |  |  | *χ ² = 157.21 p <0.001* | | |  |  |  |
|  | **Maternal and paternal education** | | | |  |  |  |  |  |  |  |  |  |
|  | Degree | 42.7 | 40.8 | 14.5 | 2 | 1360 |  | 2.3 | 18.3 | 52.5 | 26.9 | 1360 |  |
|  | A levels | 44.9 | 40.1 | 13.8 | 1.2 | 1972 |  | 2.8 | 22.5 | 53.7 | 21 | 1972 |  |
|  | O levels | 47.6 | 39.9 | 11.2 | 1.3 | 1741 |  | 4.5 | 25.4 | 52.2 | 18 | 1741 |  |
|  | Vocational/CSE | 46.7 | 39.8 | 12 | 1.6 | 1989 |  | 5.6 | 30.4 | 49.1 | 14.9 | 1989 |  |
|  |  | *χ ² = 18.40 p = 0.031* | | |  |  |  | *χ ² = 151.03 p <0.001* | | |  |  |  |
|  | **Maternal age** |  |  |  |  |  |  |  |  |  |  |  |  |
|  | 31- 44 yrs | 39.5 | 41.6 | 16.4 | 2.5 | 2277 |  | 3.1 | 21 | 49.9 | 25.9 | 2277 |  |
|  | 21 - 30 yrs | 48.1 | 40.1 | 10.9 | 1 | 4464 |  | 3.7 | 25.5 | 53.8 | 17 | 4464 |  |
|  | 20 or less | 55.1 | 29.9 | 13.7 | 1.2 | 321 |  | 12.8 | 38 | 36.4 | 12.8 | 321 |  |
|  |  | *χ ² = 100.06 p <0.001* | | |  |  |  | *χ ² = 193.65 p <0.001* | | |  |  |  |
|  | **Maternal smoking** | |  |  |  |  |  |  |  |  |  |  |  |
|  | no smoker | 47.5 | 40.2 | 11.4 | 0.9 | 5652 |  | 3.6 | 23.9 | 53.1 | 19.3 | 5652 |  |
|  | smoker | 38.3 | 39.8 | 18.3 | 3.6 | 1410 |  | 5 | 27.4 | 46.4 | 21.1 | 1410 |  |
|  |  | *χ ² = 118.78 p <0.001* | | |  |  |  | *χ ² = 23.13 p <0.001* | | |  |  |  |
|  | **Paternal smoking** | |  |  |  |  |  |  |  |  |  |  |  |
|  | no smoker | 46.9 | 40.1 | 11.9 | 1.1 | 4808 |  | 3.7 | 24.3 | 54.2 | 17.8 | 4808 |  |
|  | smoker | 43.0 | 40.1 | 14.8 | 2.1 | 2254 |  | 4.3 | 25.3 | 46.7 | 23.6 | 2254 |  |
|  |  | *χ ² = 25.2588 p < 0.001* | | | |  |  | *χ ² = 45.36 p < 0.001* | | |  |  |  |
