# Supplementary material for: Effect of Prenatal Alcohol Exposure on Childhood Academic Outcomes: Contrasting Maternal and Paternal Associations in the ALSPAC Study
Source: PLoS One. 2013 Oct 9;8(10):e74844. doi: 10.1371/journal.pone.0074844 (PMC3794033; doi:10.1371/journal.pone.0074844)
Supplement: Table S2 — Associations between maternal and paternal ‘binge’ patterns of drinking in the 1st 3 months of pregnancy and potential confounding factors (complete case analysis (n = 7062). (DOCX) [file pone.0074844.s002.docx]

**Table S2: Associations between maternal and paternal 'binge' patterns of drinking in the 1st 3 months of pregnancy and potential confounding factors (complete case analysis (n=7062)**

|  | **Maternal 'binge' patterns of drinking** | | | | | |  | **Paternal 'binge' patterns of drinking** | | | | | |
| --- | --- | --- | --- | --- | --- | --- | --- | --- | --- | --- | --- | --- | --- |
|  |  | **Never** | **1-4 days** | **5-10 days** | **10 + days** | **Total** |  | **Never** | **1-4 days** | **5-10 days** | **10 + days** | **Total** |  |
|  | **sex** | (5971) | (837) | (124) | (130) | (7062) |  | (1238) | (2659) | (n=1890) | (1813) | (7062) |  |
|  | male | 84.8 | 11.7 | 1.7 | 1.9 | 3574 |  | 17.4 | 37.3 | 26.4 | 18.9 | 3574 |  |
|  | female | 84.3 | 12 | 1.8 | 1.8 | 3488 |  | 17.7 | 38 | 24.9 | 19.4 | 3488 |  |
|  |  | *χ ² = 0.47 p = 0.93* | | |  |  |  | *χ ² = 1.93 p = 0.59* | | |  |  |  |
|  | **Marital Status** |  |  |  |  |  |  |  |  |  |  |  |  |
|  | married | 86.3 | 10.7 | 1.5 | 1.5 | 5781 |  | 17.6 | 38.4 | 25.7 | 18.2 | 5781 |  |
|  | never married | 78.5 | 16.4 | 2.4 | 2.7 | 961 |  | 17.1 | 34.7 | 25.8 | 22.5 | 961 |  |
|  | wid/separated | 70.6 | 19.1 | 5 | 5.3 | 320 |  | 16.9 | 32.8 | 25 | 25.3 | 320 |  |
|  |  | *χ ² = 103.93 p < 0.001* | | |  |  |  | *χ ² = 19.85 p = 0.003* | | |  |  |  |
|  | **Ethnicity** |  |  |  |  |  |  |  |  |  |  |  |  |
|  | white | 84.5 | 11.9 | 1.8 | 1.8 | 6955 |  | 17.2 | 37.8 | 25.9 | 19.2 | 6955 |  |
|  | other | 89.7 | 8.4 | 0 | 1.9 | 107 |  | 39.3 | 30.8 | 13.1 | 16.8 | 107 |  |
|  |  | *χ ² = 3.34 p = 0.34* | | |  |  |  | *χ ² = 37.96 p < 0.001* | | |  |  |  |
|  | **Parity** |  |  |  |  |  |  |  |  |  |  |  |  |
|  | none | 86.5 | 10.2 | 1.5 | 1.7 | 3263 |  | 15.1 | 37.2 | 27.9 | 19.7 | 3263 |  |
|  | 1-2 children | 83.2 | 13.2 | 1.9 | 1.8 | 3457 |  | 18.7 | 38.5 | 24.4 | 18.4 | 3457 |  |
|  | 3+ children | 79.5 | 14.6 | 2.9 | 2.9 | 342 |  | 28.4 | 32.7 | 17.3 | 21.6 | 342 |  |
|  |  | *χ ² = 24.04 p = 0.005* | | |  |  |  | *χ ² = 60.86 p < 0.001* | | |  |  |  |
|  | **Home mortgage/ownership** | | |  |  |  |  |  |  |  |  |  |  |
|  | yes | 86.1 | 10.9 | 1.4 | 1.6 | 5705 |  | 17.0 | 37.6 | 26.7 | 18.8 | 5705 |  |
|  | rented | 78.1 | 15.8 | 3.2 | 2.8 | 1357 |  | 20.0 | 38.0 | 21.3 | 20.8 | 1357 |  |
|  |  | *χ ² = 58.69 p < 0.001* | | |  |  |  | *χ ² = 20.62 p < 0.001* | | |  |  |  |
|  | **House crowding** | |  |  |  |  |  |  |  |  |  |  |  |
|  | <=0.5 | 87.5 | 9.5 | 1.2 | 1.7 | 3220 |  | 14.6 | 37.6 | 28.8 | 19.0 | 3220 |  |
|  | >0.5-0.75 | 82.6 | 13.3 | 2.2 | 1.9 | 3504 |  | 19.3 | 38.1 | 23.5 | 19.1 | 3504 |  |
|  | 0.75->1.0 | 76.3 | 19.5 | 1.8 | 2.4 | 338 |  | 26.9 | 33.4 | 18.3 | 21.3 | 338 |  |
|  |  | *χ ² = 55.30 p < 0.001* | | |  |  |  | *χ ² = 68.00 p < 0.001* | | |  |  |  |
|  | **Maternal and paternal education** | | | |  |  |  |  |  |  |  |  |  |
|  | Degree | 90.3 | 7.6 | 1.2 | 0.9 | 1360 |  | 18.2 | 37.8 | 27.3 | 16.7 | 1360 |  |
|  | A levels | 84.3 | 12 | 1.6 | 2.1 | 1972 |  | 15.7 | 37.3 | 26.5 | 20.5 | 1972 |  |
|  | O levels | 85.0 | 11.6 | 1.8 | 1.6 | 1741 |  | 17.3 | 36.5 | 25.4 | 20.7 | 1741 |  |
|  | Vocational/CSE | 80.5 | 14.8 | 2.3 | 2.5 | 1989 |  | 19.1 | 38.9 | 24.0 | 18.1 | 1989 |  |
|  |  | *χ ² = 62.69 p < 0.001* | | |  |  |  | *χ ² = 21.86 p = 0.009* | | |  |  |  |
|  | **Maternal age** |  |  |  |  |  |  |  |  |  |  |  |  |
|  | 31- 44 yrs | 84.8 | 11.5 | 2 | 1.7 | 2277 |  | 18.0 | 35.8 | 25.0 | 21.2 | 2277 |  |
|  | 21 - 30 yrs | 84.4 | 11.9 | 1.7 | 1.9 | 4464 |  | 16.5 | 38.9 | 26.3 | 18.2 | 4464 |  |
|  | 20 or less | 84.1 | 13.4 | 0.9 | 1.6 | 321 |  | 28.3 | 33.3 | 21.2 | 17.1 | 321 |  |
|  |  | *χ ² = 3.65 p = 0.72* | | |  |  |  | *χ ² = 41.35 p < 0.001* | | |  |  |  |
|  | **Maternal smoking** | |  |  |  |  |  |  |  |  |  |  |  |
|  | no smoker | 87.4 | 9.8 | 1.2 | 1.5 | 5854 |  | 18.3 | 38.1 | 25.5 | 18.1 | 5652 |  |
|  | smoker | 73.0 | 20.1 | 3.8 | 3.0 | 1459 |  | 14.4 | 35.9 | 26.4 | 23.3 | 1410 |  |
|  |  | *χ ² = 187.20 p < 0.001* | | |  |  |  | *χ ² = 27.81 p < 0.001* | | |  |  |  |
|  | **Paternal smoking** | |  |  |  |  |  |  |  |  |  |  |  |
|  | no smoker | 87.3 | 10 | 1.3 | 1.4 | 4808 |  | 19.0 | 39.1 | 25.7 | 16.2 | 4808 |  |
|  | smoker | 78.6 | 15.8 | 2.8 | 2.8 | 2254 |  | 14.4 | 34.5 | 25.6 | 25.5 | 2254 |  |
|  |  | *χ ² = 93.93 p < 0.001* | | |  |  |  | *χ ² = 97.53 p < 0.001* | | |  |  |  |
